# Exploring geographical differences in the incidence of colorectal cancer in the Norwegian Women and Cancer Study: a population-based prospective study

**DOI:** 10.2147/CLEP.S207413

**Published:** 2019-08-08

**Authors:** Sunday Oluwafemi Oyeyemi, Tonje Braaten, Edoardo Botteri, Paula Berstad, Kristin Benjaminsen Borch

**Affiliations:** 1 Department of Community Medicine, Uit-the Arctic University of Norway, Tromsø, Norway; 2 Department of Bowel Cancer Screening, Cancer Registry of Norway, Oslo, Norway; 3 Norwegian National Advisory Unit for Women’s Health, Women’s Clinic, Oslo University Hospital, Oslo, Norway

**Keywords:** lifestyle, diet, risk factors, colorectal cancer, women, NOWAC study

## Abstract

**Purpose:**

Norway has experienced an unexplained, steep increase in colorectal cancer (CRC) incidence in the last half-century, with large differences across its counties. We aimed to determine whether geographical distribution of lifestyle-related CRC risk factors can explain these geographical differences in CRC incidence in Norwegian women.

**Methods:**

We followed a nationally representative cohort of 96,898 women with self-reported information on lifestyle-related CRC risk factors at baseline and at follow-up 6–8 years later in the Norwegian Women and Cancer Study. We categorized Norwegian counties into four county groups according to CRC incidence and used Cox proportional hazard models to estimate hazard ratios (HRs) and 95% confidence intervals (CIs) for risk factors. We used the Karlson, Holm, and Breen (KHB) method of mediation analysis to investigate the extent to which the risk factors accounted for the observed differences in CRC incidence between counties.

**Results:**

During an average of 15.5 years of follow-up, 1875 CRC cases were diagnosed. Height (HR=1.12; 95% CI 1.08, 1.17 per 5 cm increase); being a former smoker who smoked ≥10 years (HR=1.34; 95% CI 1.15, 1.57); or being a current smoker who has smoked for ≥10 years (HR=1.28; 95% CI 1.12, 1.46) relative to never smokers was associated with increased CRC risk. Duration of education >12 years (HR=0.78; 95% CI 0.69, 0.87) vs ≤12 years, and intake of vegetables and fruits >300 g (HR=0.90; 95% CI 0.80, 0.99) vs ≤300 g per day were associated with reduced CRC risk. However, these risk factors did not account for the differences in CRC risk between geographical areas of low and high CRC incidence. This was further confirmed by the KHB method using baseline and follow-up measurements (*b*=0.02, 95% CI −0.02, 0.06, *p*=0.26).

**Conclusion:**

Lifestyle-related CRC risk factors did not explain the geographical variations in CRC incidence among Norwegian women. Possible residual explanations may lie in heritable factors.

## Introduction

Colorectal cancer (CRC) is the second most common malignancy in women globally,^1^ and the second leading cause of cancer-related death in high-income countries.^2^ Norway has experienced an unexplained, steep increase in the incidence of CRC in both men and women in the last half-century.^3^,^4^ From 1957–61 to 2012–16, incidence rates among Norwegian women increased from 21 to 54 per 100,000 person-years for colon cancer, and from 9 to 20 per 100,000 person-years for rectal cancer.^5^ The CRC incidence rates among women in Norway are currently among the highest in the world,^6^ having almost tripled from 1957–61 to 2012–16, and surpassing the rates in other Nordic countries with apparently similar lifestyles. So far, the reasons for this steep increase have been elusive. Moreover, differences in CRC incidence vary over 10-fold across countries,^7^ which may be ascribed to variations in dietary and environmental exposures, coupled with genetic susceptibility.^8^ CRC incidence also varies within Norway, with a more than 20 per 100,000 person-years difference between areas of high and low CRC incidence.^9^,^10^ The factors responsible for this geographical heterogeneity are yet to be determined, and knowledge of these factors could be useful to guide screening strategies and health policy.

Therefore, this study aimed to determine whether the geographical distribution of lifestyle-related CRC risk factors can explain the geographical differences in CRC incidence, using the Norwegian Women and Cancer (NOWAC) Study.

## Materials and methods

The NOWAC Study is a nationwide, representative prospective cohort study which started in 1991.^11^ The full detail of the cohort profile has been described previously.^11^,^12^ Summarily, the study consists of over 172,000 women who were recruited over three different time periods: 1991–92, 1996–97, and 2003–04. Potential participants aged 30–70 years were randomly selected from the Norwegian Central Population Register (Statistics Norway) and received a questionnaire by mail that collected information on their lifestyle and health status at enrollment (baseline questionnaire). Similar follow-up questionnaires were sent to the same women about 6–8 years later. All women who agreed to participate completed and returned the questionnaires with written informed consent. The NOWAC Study was approved by the Regional Committee for Medical Research Ethics and the Norwegian Data Inspectorate.^11^

NOWAC participants who were enrolled in 1991–92, 1996–97, and 2003–04 and completed a food frequency questionnaire (FFQ) in 1998, 1996–97, and 2003–04, respectively, were eligible for inclusion in the present study. Those who were enrolled in 1991–92 completed an FFQ in 1998 because an FFQ was not included in the 1991–92 questionnaire. Thus, we used the 1998 information as baseline for the participants enrolled in 1991–92. This represented 101,321 participants who completed a baseline questionnaire with dietary information between 1996 and 2004. We subsequently excluded women who died or emigrated (n=14) prior to the start of follow-up, and all cases of prevalent cancer except non-melanoma skin cancer (n=4,414). This resulted in a final study sample of 96,893 women. Follow-up information was available for 68,626 (70.8%) of these women.

### Assessment of CRC risk factors

Information on age, physical activity, height, weight, duration of education, alcohol intake, smoking status and intensity (pack-years), annual household income, hormone replacement therapy use, oral contraceptive use, and dietary habits (daily intake of red meat, processed meat, fish, fruits and vegetables, fiber, calcium, vitamin D, and milk) were taken from the NOWAC questionnaire. Physical activity was reported on a validated 10-point scale, on which 1 was “very low” and 10 was “very high”. This is a global (ie, all-inclusive) physical activity score that has been found valid to rank the physical activity of women in the NOWAC Study.^13^ The validated, self-reported height and weight measurements from the questionnaires were used to compute body mass index (BMI).^14^ Information on the duration of education and alcohol intake was obtained from the questionnaire, while information on smoking status and smoking intensity (pack-years) were combined into one variable of smoking history. Information on annual household income, hormone replacement therapy use, and oral contraceptive use were also extracted from the NOWAC questionnaire. The FFQ includes foods that are common in Norway and has been validated.^15^,^16^

The choice of these CRC risk factors was based on the literature, previous similar studies,^8^,^17^ and the availability of information in the NOWAC Study.

### Assessment of county of residence and creation of county groups by CRC incidence

**Figure 1:**
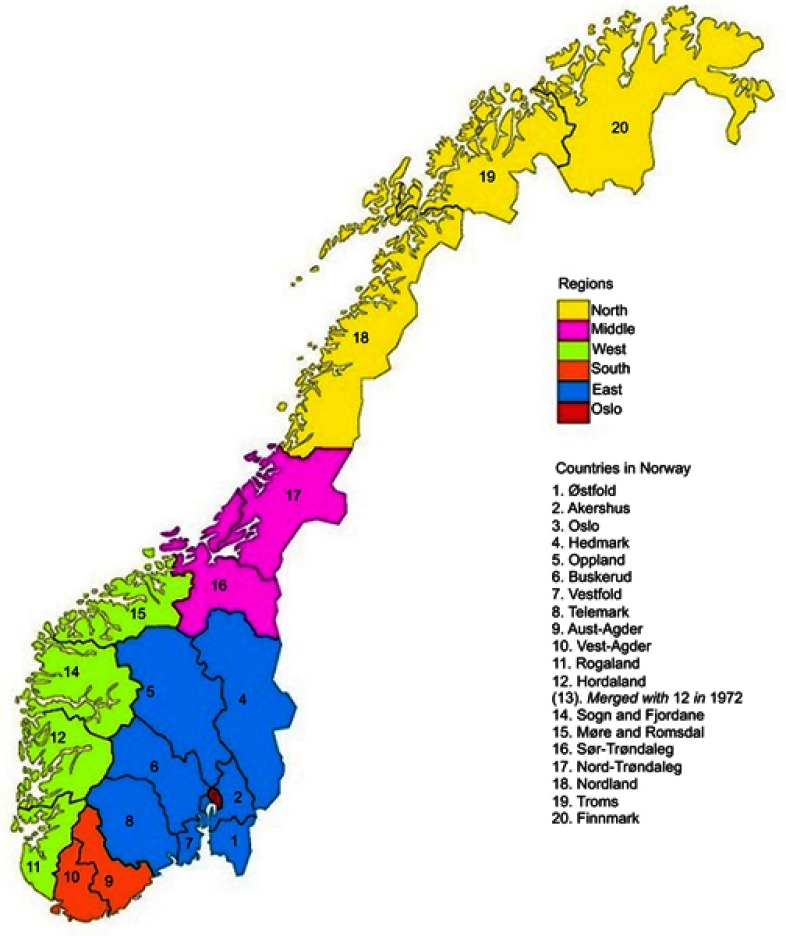
Map of Norway showing the 19 counties and regions.

County of residence at baseline was accessed through linkage to the Norwegian Central Population Register (Statistics Norway). There were 19 counties in Norway at the time of data collection (Figure 1). We used percentiles of CRC incidence rate (Table 1) to categorize the counties into four groups. The intent was to compare the lowest 10% to the highest 10% to discern possible differences in lifestyle-related CRC risk factors. However, we raised the limit of the low-incidence counties to the 15th percentile to allow for more cases of CRC in this group. Thus, we grouped counties from 0 to 15th percentile as low-incidence counties (Oppland, Sør Trøndelag, and Telemark); 15–50th as mid-low-incidence counties (Hedmark, Hordaland, Oslo, Møre and Romsdal, Nord-Trøndelag, Vest-Agder, and Buskerud); 50–90th as mid-high-incidence counties (Rogaland, Akershus, Aust-Agder, Vestfold, Østfold, Finnmark, and Troms); and 90–100th as high-incidence counties (Nordland, Sogn and Fjordane).

**Table 1:** Basic parameters and endpoints in the 19 counties of Norway in the Norwegian Women and Cancer Study

Counties	Sample population per county	Number of CRC cases	Incidence proportion of CRC (%)	Crude incidence rate per 100,000	Average follow-up time in years	Person-years at risk
Østfold	4836	106	2.2	146	15.0	72,563
Akershus	9661	177	1.8	121	15.1	146,259
Oslo	8439	142	1.7	111	15.1	127,573
Hedmark	3808	62	1.6	108	15.2	57,671
Oppland^a^	3544	47	1.3	88	15.0	53,315
Buskerud	4496	78	1.7	115	15.1	67,970
Vestfold	4267	81	1.9	125	15.2	64,808
Telemark	3137	45	1.4	96	15.0	46,975
Aust-Agder	1827	34	1.9	123	15.1	27,640
Vest-Agder	2715	47	1.7	114	15.1	41,088
Rogaland	6503	117	1.8	119	15.2	98,500
Hordaland	7736	130	1.7	110	15.2	117,863
Sogn og Fjordane^b^	1889	49	2.6	171	15.2	28,655
Møre og Romsdal	4653	80	1.7	112	15.3	71,354
Sør Trøndelag	4882	67	1.4	91	15.1	73,835
Nord-Trøndelag	2607	45	1.7	114	15.2	39,530
Nordland	11,443	322	2.8	169	16.7	190,621
Troms	7264	176	2.4	146	16.6	120,723
Finnmark	3186	70	2.2	132	16.7	53,171
Total	96,893	1,875	1.9	125	15.5	1,500,112

**Notes:** ^a^County with lowest CRC incidence. ^b^County with highest CRC incidence.

**Abbreviation:** CRC, colorectal cancer.

We also conducted sensitivity analyses in which, we grouped participants by region of residence (Oslo, East, South, West, Middle, and North)^18^ and by rural/urban area of residence. Urban residence was defined as living in a “dense area” with a maximum distance of 50 m between houses, except for public areas or natural barriers, and inhabited by at least 200 persons.^19^

### CRC incidence, emigration, and death

Participants diagnosed with primary colon or rectal cancer were ascertained through linkage to the Cancer Registry of Norway. We used the International Statistical Classification of Diseases and Related Health Problems, Tenth Edition (ICD-10), which uses code C18 for colon and C19-20 for rectal cancer. The county of residence, date of emigration, and date of death were ascertained via linkage to the Norwegian Central Population Register (Statistics Norway).

### Analytic variables

We carried out an initial analysis using the baseline data to assess the CRC risk factors for multi-collinearity. This initial analysis included height (continuous, in meters); physical activity (dichotomized into inactive (1–5) and active (6–10)); BMI (<20.0, 20.0–24.9, 25.0–29.9, and ≥30.0 kg/m^2^); duration of education (≤12 and >12 years); alcohol intake (0, ≤3.0, >3.0–10.0, and >10.0 g/day); smoking history (never, former smoker of <10 years, former smoker of ≥10 years, current smoker of <10 years, current smoker of ≥10 years); annual household income in Norwegian kroner (NOK) (low: <300,000 NOK, medium: 300–600,000 NOK, and high: >600,000 NOK); hormone replacement therapy use (never/ever); and oral contraceptive use (never/ever). All the dietary variables were dichotomized along their median values: red meat intake (0, ≤15, >15 g/day); processed meat intake (0, ≤70, >70 g/day); fish intake (0–90, >90 g/day); fruit and vegetable intake (0–300, >300 g/day); fiber (0–21, >21 g/day); calcium intake from food (0–700, >700 mg/day); vitamin D intake (0–6, >6 µg/day); and milk intake (0, ≤170, >170 g/day). Where possible, we used the median values (50th percentile) to split the variables into categories, as the median values are more robust and undistorted by outliers.^20^

### Statistical methods

We present descriptive statistics at baseline as mean values (±standard errors, SEs) or percentages. We used Cox proportional hazard regression models with age as the time scale to estimate the hazard ratios (HRs) and 95% confidence intervals (CIs) for the associations between the county groups (low-, mid-low-, mid-high-, and high-incidence counties), risk factors, and CRC incidence. Follow-up time was defined as the period in years between age at baseline and age at diagnosis of incident cancer, death, emigration, or age at the end of follow-up (31 December 2016), whichever came first.

We assessed predefined possible interaction effects between physical activity versus BMI, smoking history, alcohol intake, and dietary factors, respectively. We also checked for interaction effects between duration of education and BMI, smoking history, alcohol intake, and dietary factors, respectively. We tested for multi-collinearity between calcium versus milk and vitamin D intake, respectively; red meat versus processed meat intake; and fiber versus fruit and vegetable intake. We excluded milk because of high collinearity with calcium and >25% missing values in the variable. We repeated the baseline analyses following exclusion of cancers diagnosed in the first 2 years of follow-up to control for possible reverse causality. Sensitivity analyses were carried out by region of residence, and area of residence (rural/urban).

### Mediation analysis using Karlson, Holm, and Breen (KHB) method of decomposition

We used the KHB method of mediation analysis^21^ to investigate the extent to which the CRC risk factors (mediating variables) account for the observed difference in CRC incidence between individual counties. The KHB method provides decomposition of the total effects of counties on CRC incidence into direct and indirect effects.^21^ The basic outputs from the KHB method include three models: the reduced model, the full model, and the difference (model). The reduced model describes the estimated effect of the counties with no mediating variables in the model (total effect). The full model describes the estimated effect of counties with all mediating variables in the model (direct effect). The difference between these two models represents the indirect effect. The indirect effect is interpreted as the mediation effect. The KHB method assumes a normal distribution of the indirect effect, and this assumption has been shown to be legitimate in large samples such as the NOWAC Study.^22^ We fitted the KHB models using the data collected at baseline and then used the multiply imputed data.

### Multiple imputation and repeated measurements analyses

Multiple imputation using chained equations was used to handle missing data, under the assumption that this data was missing at random.^23^ The missing values were replaced by multiply imputed values from 20 duplicate datasets. We created 20 duplicates datasets from the imputation simulation to reduce sampling variability.^24^ We included all the CRC risk factors used in the analyses and the Nelson–Aalen cumulative hazard estimator as predictors in the imputation model.^25^,^26^ We used Rubin’s rules to combine the estimates from the 20 imputed datasets to estimate HRs and corresponding 95% CIs.^27^ The KHB method also computes the total, direct, and indirect effects for each imputed dataset and combines the estimates using Rubin’s rules.

We used baseline information up to the point when follow-up information was available on physical activity, BMI, alcohol intake, smoking history, hormone replacement therapy use, and all dietary intakes. We then used the follow-up information until death, emigration, or the end of the study, whichever occurred first.

All the analyses and multiple imputations were done in Stata version 15.0 (StataCorp, College Station, TX, USA). Figure 1 is produced using GraphPad Prism 8 (GraphPad Software, San Diego, CA). All statistical analyses were two-sided, and *p*-values were considered statistically significant at a level of <0.05.

## Results

During an average of 15.5 years of follow-up and 1.5 million person-years, 1875 CRC cases (1276 [68%] colon cancers and 599 [32%] rectal cancers) were diagnosed in the study sample. The counties of lowest and highest crude incidence rates were Oppland, and Sogn and Fjordane, respectively (Table 1).

The median age at baseline was 51 years, while the median age at diagnosis of CRC was 66 years (range 43–89). When looking at county groups, low-incidence counties had a higher proportion of physically active women compared to high-incidence counties (46% vs 41%) at baseline. Similarly, the low-incidence counties had a higher proportion of women with a longer duration of education (38% vs 25%), never smokers (38% vs 34%), high annual household income (12% vs 5%), hormone replacement therapy use (34% vs 30%), and oral contraceptive use (53% vs 43%), compared to high-incidence counties. Conversely, high-incidence counties had higher proportion of women with overweight (33% vs 31%), obese (10% vs 9.6%), ever smokers (64% vs 60%), and low annual household income (48% vs 36%), compared to low-incidence counties (Table 2).

**Table 2:** Selected participant characteristics by county group at study enrollment (baseline) in the Norwegian Women and Cancer Study

Characteristics	Low incidence: (Oppland, Sør-Trøndelag, Telemark)	Mid-low incidence: (Hedmark, Hordaland, Oslo, Møre and Romsdal, Nord-Trøndelag, Vest-Agder, Buskerud)	Mid-high incidence: (Rogaland, Akershus, Aust-Agder, Vestfold, Østfold, Troms, Finnmark)	High incidence: (Nordland, Sogn and Fjordane)
Population	11,563	34,454	37,544	13,332
Colorectal cancer, n (% in the area)	159 (1.4)	584 (1.7)	761 (2.0)	371 (2.8)
Crude incidence rate per 100,000	91	112	130	169
Mean age at baseline in years	51.6	51.6	52.1	53.7
Physical activity (% active, 6–10)	46	46	44	41
Mean height in cm (SE)	166 (0.05)	167 (0.03)	166 (0.03)	165 (0.05)
Mean body mass index (SE)	24.9 (0.04)	24.6 (0.02)	24.8 (0.02)	25.1 (0.03)
Mean duration of education in years (SE)	12.2 (0.03)	12.5 (0.02)	12.0 (0.02)	10.9 (0.03)
Mean alcohol intake in g/day (SE)	3.5 (0.04)	3.9 (0.03)	3.6 (0.02)	2.6 (0.03)
Smoking history, %				
Never	38	38	37	34
Former	31	32	32	32
Current	29	28	30	32
(% of ever smokers)	(60)	(60)	(62)	(64)
Annual household income, %				
Low (<300,000 NOK)	36	33	35	48
Medium (300,000–600,000 NOK)	45	45	443.6	38
High (>600,000 NOK)	12	15	14	5
Hormone therapy use (% of ever users)	34	35	33	30
Oral contraceptive use (% of ever users)	53	55	52	43
Dietary factors				
Mean red meat intake in g/day (SE)	14.3 (0.10)	14.8 (0.06)	15.4 (0.06)	15.0 (0.10)
Mean processed meat in g/day (SE)	68.9 (0.38)	69.1 (0.23)	68.6 (0.21)	60.8 (0.33)
Mean fish intake in g/day (SE)	87.2 (0.50)	92.4 (0.30)	96.9 (0.31)	121.0 (0.60)
Fruit and vegetables intake in g/day (SE)	337 (1.9)	349 (1.1)	333 (1.0)	292 (1.6)
Mean fiber intake in g/day (SE)	21.2 (0.08)	21.5 (0.04)	20.9 (0.04)	20.6 (0.06)
Calcium intake in mg/day (SE)	745 (3.4)	740 (1.6)	744 (1.7)	730 (2.7)
Vitamin D in μg/day (SE)	8.56 (0.08)	8.65 (0.04)	9.10 (0.04)	9.36 (0.07)

**Abbreviation:** SE, standard error.

The variables with the highest proportion of missing values at baseline were physical activity (9.5%), annual household income (7.3%), and duration of education (5.8%). At follow-up, 38% of the women had missing values on physical activity, and approximately 30% had missing information on BMI, alcohol intake, smoking history, hormone replacement therapy use, and dietary intakes. There was no substantial change in the characteristic features of the study sample between the imputed and the complete-case dataset (Table S1).

**Figure 2:**
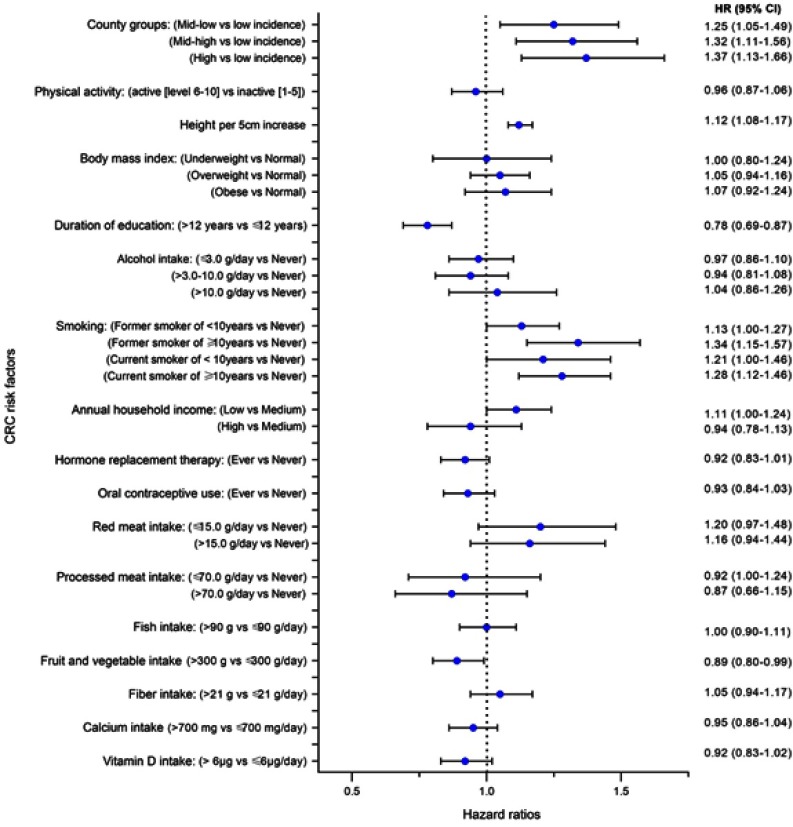
Multivariable hazard ratios (HRs) and 95% confidence intervals (CIs) of factors associated with colorectal cancer (CRC) incidence at baseline and follow-up with chained multiple imputations, in the Norwegian Women and Cancer study.

The multivariable-adjusted model of repeated measurements showed that the high-incidence county group had an HR of 1.37 (95% CI 1.13–1.66) relative to the low-incidence county group (Figure 2), which was similar to the unadjusted estimate (Table S2). Height (HR=1.12; 95% CI 1.08, 1.17 per 5 cm increase), being a former smoker who smoked ≥10 years (HR=1.34; 95% CI 1.15, 1.57), or a current smoker who had been smoking ≥10 years (HR=1.28; 95% CI 1.12, 1.46), compared to never smokers, were significantly associated with a higher CRC risk. Duration of education >12 years (HR=0.78; 95% CI 0.69, 0.87) compared to ≤12 years, and daily fruit and vegetable intake >300 g (HR=0.90; 95% CI 0.80, 0.99) compared to ≤300 g, were associated with decreased CRC risk (Figure 2).

No substantial difference was seen after excluding those who were diagnosed with CRC during the first 2 years of follow-up (data not shown). Sensitivity analyses by region showed no differences in the HR estimates for CRC risk factors, nor were any statistically significant differences seen in the HR estimates for the regions before and after multivariable adjustment. This was also the case in sensitivity analyses that used rural/urban area of residence (Table S2).

The KHB analysis showed the extent to which the mediating variables (CRC risk factors) account for the difference in CRC incidence between the low-incidence county group (reference) and that of other county groups. At baseline, the log odds of having CRC in the high-incidence county group were 0.41 higher than those in the low-incidence county group (Table 3). After adjusting for mediating factors, the effect of living in the high-incidence county group reduced to 0.39, leaving an indirect effect of 0.02 (*b*=0.02; 95% CI −0.02, 0.06, *p*=0.26). This shows that the differences in CRC incidence between the low- and high-incidence county groups are not significantly mediated by the combined effects of the investigated CRC risk factors (Table 3). The mediation analysis results in the imputed dataset were similar to the baseline results. We conducted a sensitivity analysis using the 19 counties individually (without grouping), which also showed that the combined effects of the risk factors did not significantly mediate the variations in CRC incidence across counties (data not shown).

**Table 3:** Decomposition of total effects of county groups into direct and indirect effects using the Karlson, Holm, and Breen method at baseline and follow-up in the Norwegian Women and Cancer Study

County groups		Baseline data		Imputed data	
		Coefficient (95% CI)	*p*-value	Coefficient (95% CI)	*p*-value
Low incidence	(base outcome)	–	–	–	–
Mid-low incidence					
	Reduced model	0.252 (0.040, 0.463)	0.020	0.198 (0.021, 0.375)	0.028
	Full model	0.253 (0.041, 0.465)	0.019	0.205 (0.028, 0.383)	0.023
	Difference	−0.001 (−0.018, 0.016)	0.880	−0.007 (−0.022, 0.007)	0.316
Mid-high incidence					
	Reduced model	0.317 (0.109, 0.526)	0.003	0.268 (0.095, 0.442)	0.002
	Full model	0.321 (0.113, 0.530)	0.003	0.277 (0.103, 0.451)	0.002
	Difference	−0.004 (−0.024, 0.016)	0.690	−0.009 (−0.026, 0.008)	0.228
High incidence					
	Reduced model	0.409 (0.175, 0.642)	0.001	0.342 (0.150, 0.535)	<0.001
	Full model	0.388 (0.152, 0.624)	0.001	0.323 (0.129, 0.518)	0.001
	Difference	0.021 (−0.016, 0.057)	0.263	0.019 (−0.013, 0.048)	0.253

**Abbreviation:** CI, confidence interval.

## Discussion

In this large cohort of Norwegian women, we found that county-level differences in CRC incidence were not explained by differences in lifestyle-related CRC risk factors. This was demonstrated by two different approaches: Cox proportional hazards models and the relatively new KHB method of decomposition.

The lifestyle-related CRC risk factors significantly associated with CRC incidence in our cohort of women included height, smoking history, duration of education, and fruit and vegetable intake. Our results showed that these factors, together with other CRC risk factors, did not significantly explain the differences in the CRC incidence between the counties. CRC risk in county groups remained statistically the same before and after adjusting for risk factors. These results remained consistent when using baseline data, as well as when using repeated measurements with multiple imputation. Our findings suggest that there are other important or unmeasured risk factors that are responsible for the differences in CRC incidence between Norwegian counties.

Previous international studies have rationalized that variations in CRC incidence in different areas of a country are due to different, but overlapping, contributory factors, such as rural–urban disparities, socioeconomic status (SES), ease of access to health care, public health campaigns, unique social and lifestyle risk factors, differences in exposure to risk factors, such as in dietary customs and ethnic variations in food preparation, and different exposures to unknown risk factors.^28^–^31^ Some studies have indicated that rural–urban disparities confer an increased risk of CRC in rural areas^32^,^33^ and suggested that the relationship may be mediated through screening behavior.^32^,^33^ Other studies have reported that the increased risk may simply reflect the socioeconomic differences between rural and urban communities.^34^ Other studies found a higher risk of CRC in urban areas.^34^–^36^ These findings differ by country and time period of assessment, and differences in the definition of rural/urban areas may mask the relationship between this variable and CRC risk.^35^ There is currently no national CRC screening program in Norway, which could expound on some of the geographical differences in the present population.

Education and household income are often used as proxy indicators of SES. We found a significant inverse association between duration of education and CRC risk, while we found no such association with annual household income. Results of previous similar studies regarding SES have been inconsistent. A recent review showed that, in the United States and Canada, low SES groups have a higher CRC incidence than high SES groups (RR from 1.0 to 1.5), while these findings were mostly reversed (RR from 0.3 to 0.9) in Europe.^30^ Nonetheless, education, and not necessarily income, may be a better predictor of a healthy lifestyle.^37^,^38^

Cigarette smoking has been associated with increased incidence of CRC, and our data further suggest that the risk remains even among former smokers. A meta-analysis of 106 observational studies concluded that smokers have an increased risk of developing CRC compared to never smokers (RR 1.18, 95% CI 1.11–1.25).^39^ Height was also associated with increased CRC risk in our study sample. This finding is in agreement with two recent systematic reviews of prospective studies, which posited a potential causal association of adult attained height with the risk of CRC.^40^,^41^ Our study found a significant inverse association between fruit and vegetable intake and CRC risk, which is in concurrence with the findings in the European Prospective Investigation into Cancer and Nutrition (EPIC) study.^42^

In our study, participants in the low-incidence county group were more physically active, had a longer duration of education, were more often never smokers, and had a higher fruit and vegetable intake. These are markers of a generally healthy lifestyle, and the reduced CRC risk observed in this county group may be a reflection of this lifestyle. Notwithstanding, these factors failed to account for the risk differences between low- and high-incidence county groups.

Occurrence of exposure to established risk factors for cancer has been reported to vary geographically within some countries. For instance, the prevalence of obesity varies within Finland,^43^ while the use of hormone replacement therapy is more likely in women living in urban areas of Denmark.^44^ Therefore, it is plausible that the risk of CRC could vary in different counties or areas due to different prevalences of exposure to established CRC risk factors. However, since these established risk factors did not account for the observed risk differences in CRC between the counties in the present study, considerable uncertainty remains about what is responsible for these differences. This may be a partial reflection of the incomplete understanding of the carcinogenesis of CRC,^34^ although the unexplained risk differences could also come from unmeasured risk factors. A large Scandinavian study, which combined cohorts of twins from Sweden, Denmark, and Finland, demonstrated that genetically inheritable factors account for 35% of the CRC cases, while non-shared environmental factors account for 60%, and shared environmental factors the remaining 5%.^45^ Thus, a possible explanation for our observed differences in risk between high- and low-incidence county groups probably lies more in genetically inherited factors. The well-described CRC-related inheritable syndromes (such as hereditary nonpolyposis colon cancer (HNPCC) and familial adenomatous polyposis (FAP)), where inheritance is highly penetrant, only account for about 3–5% of the inherited cases of CRC.^46^

The main limitations of this study are the unmeasured established CRC risk factors. This includes family history of CRC and its precursors (such as adenomatous polyps), as genetically inherited factors can increase the likelihood of CRC oncogenesis.^45^,^46^ Our study lacks information on the use of aspirin and other non-steroidal anti-inflammatory drugs, the regular use of which has been associated with reduced CRC risk.^47^,^48^ The lack of information on these factors may have confounded our study. The county of residency used in this study was captured only at baseline; thus, some of the participants could have changed their county of residence in the course of the study. However, most women at the age of our cohort would have settled down at a county on a long-term basis. We lack the power to explore the CRC risk in each county or in each county group separately. Most variables in our study are self-reported and therefore are saddled with the errors inherent with self-reported measurements. However, most of these variables, such as physical activity, duration of education, BMI, alcohol intake, and dietary habits, have been validated with good results.^12^–^16^

The strengths of our study include the prospective and population-based design, with a large sample size of participants who were randomly recruited and are representative of Norwegian women between 30 and 70 years at recruitment,^12^ information on important risk factors, and the high quality of the national cancer registry with almost 100% completeness.^49^ The NOWAC Study has been shown to have almost the same observed cumulative incidence rates for all cancer sites as that of the national figures.^11^,^12^ We used repeated measurements of variables to account for changes in these variables over time in order to lower the risk of measurement error. We used chained multiple imputation to deal with missing data, and thus maximize the number of participants, and by extension, the number of CRC cases included in the analyses.

## Conclusion

The lifestyle-related CRC risk factors that we investigated did not account for the risk differences between the areas of low and high incidence of CRC. A possible explanation lies in inheritable factors. Thus, the family history of CRC cases may be especially important in determining the appropriate preventive screening strategy in areas of high incidence.

## Supplementary materials

**Table S1:** Comparison of the complete-case and imputed dataset, the Norwegian Women and Cancer study

Characteristics		Missing n (%)	Complete-case mean (SD), or %	Multiply imputed mean (SD), or %
County of residence		0 (0)		
	Low incidence (%)		12	12
	Mid-low incidence (%)		36	36
	Mid-high incidence (%)		39	39
	High incidence (%)		14	14
Age at baseline (SD)		0 (0)	52.1 (6.7)	52.1 (6.7)
Physical activity (SD)		9,214 (9.5)	5.6 (1.8)	5.5 (1.8)
Height (SD)		561 (0.6)	166.1 (5.7)	166.1 (5.7)
Body mass index (SD)		2,187 (2.3)	24.8 (4.0)	24.8 (4.0)
Duration of education (SD)		5,601 (5.8)	12.1 (3.5)	12.0 (3.5)
Alcohol intake (SD)		1,958 (2.0)	3.6 (4.5)	3.5 (4.5)
Smoking status (%)		1,869 (1.9)		
	Never (%)		37	37
	Ex (%)		33	33
	Current (%)		30	30
Pack years (SD)		6 (0.01)	6.3 (8.5)	6.3 (8.5)
Annual household income		7,054 (7.3)		
	Low (%)		39	39
	Medium (%)		47	47
	High (%)		14	14
Hormone replacement therapy use		2,793 (2.9)		
	Never (%)		66	66
	Ever (%)		34	34
Oral contraceptive use		3,695 (3.8)		
	Never (%)		54	53
	Ever (%)		46	47

**Abbreviation:** SD, standard deviation.

**Table S2:** Hazard ratios (HRs) and 95% confidence intervals (CIs) before and after multivariable risk adjustment at baseline (complete-case analysis) in the Norwegian Women and Cancer study

Groupings by		Baseline (complete-case analysis)		Baseline and follow-up (with chained multiple imputation)	
		Crude (unadjusted)^a^	Multivariable adjusted^b^	Crude (unadjusted)^a^	Multivariable adjusted^b^
	Categories	HR (95% CI)	HR (95% CI)	HR (95% CI)	HR (95% CI)
CRC incidence	Low	1.00	1.00	1.00	1.00
	Mid-low	1.29 (1.04–1.59)	1.29 (1.05–1.59)	1.22 (1.03–1.46)	1.25 (1.05–1.49)
	Mid-high	1.37 (1.12–1.69)	1.39 (1.13–1.71)	1.30 (1.10–1.55)	1.32 (1.11–1.56)
High	1.51 (1.20–1.89)	1.52 (1.20–1.92)	1.41 (1.17–1.71)	1.37 (1.13–1.66)
Regions	Oslo	1.00	1.00	1.00	1.00
	East	1.03 (0.84–1.26)	1.05 (1.85–1.29)	1.06 (0.88–1.27)	1.03 (0.86–1.24)
	South	1.08 (0.79–1.48)	1.10 (0.80–1.51)	1.08 (0.82–1.41)	1.04 (0.79–1.37)
	West	1.12 (0.90–1.40)	1.18 (0.94–1.48)	1.08 (0.89–1.31)	1.07 (0.88–1.30)
	Middle	0.83 (0.62–1.11)	0.85 (0.64–1.14)	0.91 (0.71–1.16)	0.87 (0.68–1.12)
North	1.04 (0.84–1.28)	1.05 (0.84–1.32)	1.03 (0.85–1.24)	0.95 (0.78–1.16)
Rural–urban area of residence	Rural	1.00	1.00	1.00	1.00
Rrban	1.04 (0.94–1.16)	1.05 (0.94–1.17)	1.03 (0.94–1.12)	1.05 (0.96–1.15)

**Notes:** ^a^Unadjusted except for age (age was used as the time scale). ^b^Adjusted for age, physical activity, height, body mass index, duration of education, alcohol intake, smoking history, hormone replacement therapy use, oral contraceptive use, annual household income, and dietary factors.

## Data Availability

To access the data supporting the findings presented, kindly contact the person in charge of the NOWAC Study - https://site.uit.no/nowac/contact-information/.

## Abbreviations

CRC, colorectal cancer; NOWAC, Norwegian Women and Cancer Study; FFQ, Food frequency questionnaire; BMI, body mass index; NOK, Norwegian kroner; KHB method, Karlson, Holm, and Breen method.
